# Origin of the Shape of Current-Voltage Curve through Nanopores: A Molecular Dynamics Study

**DOI:** 10.1038/srep25750

**Published:** 2016-05-11

**Authors:** Takashi Sumikama

**Affiliations:** 1 University of Fukui, Faculty of Medical Sciences, Fukui, Japan

## Abstract

Ion transports through ion channels, biological nanopores, are essential for life: Living cells generate electrical signals by utilizing ion permeation through channels. The measured current-voltage (*i-V*) relations through most ion channels are sublinear, however, its physical meaning is still elusive. Here we calculated the *i-V* curves through anion-doped carbon nanotubes, a model of an ion channel, using molecular dynamics simulation. It was found the *i-V* curve reflects the physical origin of the rate-determining step: the *i-V* curve is sublinear when the permeation is entropy bottlenecked, while it is superlinear in the case of the energy bottlenecked permeation. Based on this finding, we discuss the relation between the molecular mechanism of ion permeation through the biological K^+^ channels and the shape of the *i-V* curves through them. This work also provides a clue for a novel design of nanopores that show current rectification.

A nanopore is a molecule-sized hole equivalent to a nanometer in size^1^. Examples of nanopores in the fields of material science are carbon nanotubes (CNTs), zeolites, and so on. The transports of polymers, water, ions, and proton through them are applied to molecular sensors and gene deliveries^2^,^3^,^4^,^5^. Compared with this recent active field, ion channels, biological nanopores, have a long history^6^. They are membrane proteins that transmit electrical signals in muscles and nervous systems by passively transporting ions under the electrochemical field^7^. It was found that they have a narrow pore as an ion permeation pathway, where ions and water molecules align in a single-file^6^,^8^.

The current through a single channel molecule (*i*), measured by electrophysiological techniques such as the patch-clamp method^9^, has been evaluated to explore the permeation mechanism. Most ion channels show non-ohmic *i-V* curves, where *V* is the applied voltage: the *i-V* curves are usually sublinear, that is, *i* is saturated at a high *V*. This saturation means that the ion permeation rate is limited by the process which cannot be accelerated by *V*, and has been historically considered to be a sign of diffusion-limited ion permeation; ion diffusion to the entrance of channels controls the ionic current^7^,^10^. A simple estimate, however, clarified that the permeation is diffusion-limited when the channel conductance is beyond ~300 pS, well above the upper limit of most channels (~50 pS)^7^. Hence, the diffusion limited permeation does not occur under usual or physiological condition, thus the physical meaning of the sublinear *i-V* curve still remains unclear.

One of the tools that can elucidate the atomistic mechanism of the permeation process is the molecular dynamics (MD) simulation. Since the determination of the x-ray crystal structure of the K^+^ channel^8^, several studies using the MD simulations observed K^+^ permeation through the K^+^ channels^11^,^12^,^13^,^14^,^15^,^16^. Among them, one study calculated the *i-V* curve through the K^+^ channel:^15^ unfortunately, the calculated *i* was rather smaller than the experimental value and the *i-V* curve was superlinear, resulting in no current less than the physiological voltage (<200 mV). An investigation of the failure of this advanced technique or an analysis of the meaning of different shapes of the *i-V* curves is thus an immediate issue to be addressed.

In our previous study, the ion permeation through an anion-doped carbon nanotube (ANT), a model of the narrowest part of the K^+^ channel, was investigated using the MD simulation^17^,^18^. This simplification enabled us to perform many trajectory calculations. Several kinds of charge on the ANT were examined. It was found that, regardless of the charge, only about 10% of ions that approached the channel entrance really entered, that is, the transport through the ANT was not diffusion limited^17^. In the case of ANT^−5^ (the superscript indicates the charge on ANT hereafter), the entry of ions was hindered by a water molecule located at the entrance, and this hindrance causes a free energy barrier determining the permeation rate^17^. This free energy barrier was found to be made by the entropy bottleneck due to the narrow phase space for the exchange of the water molecule located at the entrance and an incoming ion^18^. On the other hand, in the case of ANT^−6^, the permeation was found to be energy bottlenecked mainly because of the ion-ion repulsion. It is thus extremely important to examine the *i-V* curves through ANTs to find the relation between the physical origin and its shape.

## Results and Discussion

### The relevance between the shape of the current-voltage curves and the physical origin of the rate-limiting step

**Figure 1:**
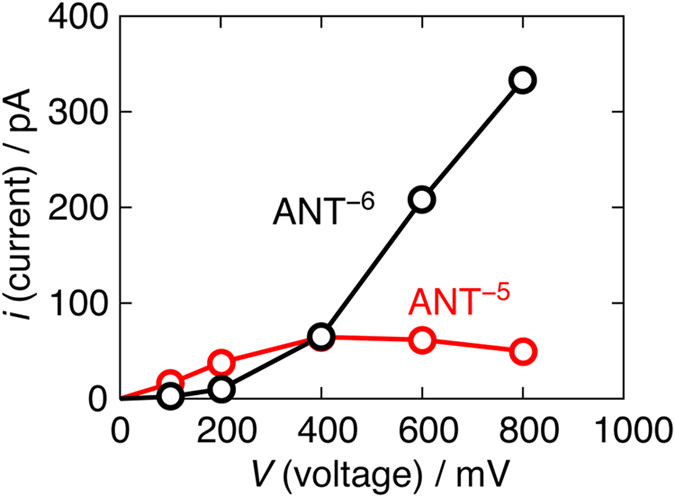
The current-voltage (*i-V*) curves through ANT^−5^ and ANT^−6^. The red line is the *i-V* curve through ANT^−5^, and the black for ANT^−6^. The *i-V* curve through ANT^−5^ is sublinear, while that through ANT^−6^ is superlinear.

The *i-V* curves through the ANTs were obtained by the MD simulation and are shown in Fig. 1. The red line is that through ANT^−5^, and the black ANT^−6^. Note that *i* through ANT^−5^ and ANT^−6^ is ~720 pA (=*eN*_appr_, where *N*_appr_ is the ion approaching rate to the entrance), when all the ions arrived at the entrance permeate, that is, in the case of diffusion limitation^17^.

The *i-V* curve through ANT^−5^ is sublinear, in which *i* is saturated at 400 mV. This saturation behavior is identical to those of most biological channels. Recall that the narrowness or the limited pathway of the phase space for exchanging the coordination determines the ion transport rate through ANT^−5^. When this pathway for the replacement does not increase as *V* increases, the free energy barrier does not decrease, leading to the saturation of the permeation rate.

On the other hand, the *i-V* curve through ANT^−6^ is superlinear. This behavior is similar to that through the K^+^ channel obtained by the MD simulation^15^. For ANT^−6^, the ion-ion repulsion mostly controls the permeation rate^18^. The shape of the *i-V* curve means that the force arisen from the electric field compensates the repulsive force between ions. It leads to the lower energy barrier, and so the lower free energy barrier. Thus, the current superlinearly increases when the voltage increases.

**Figure 2:**
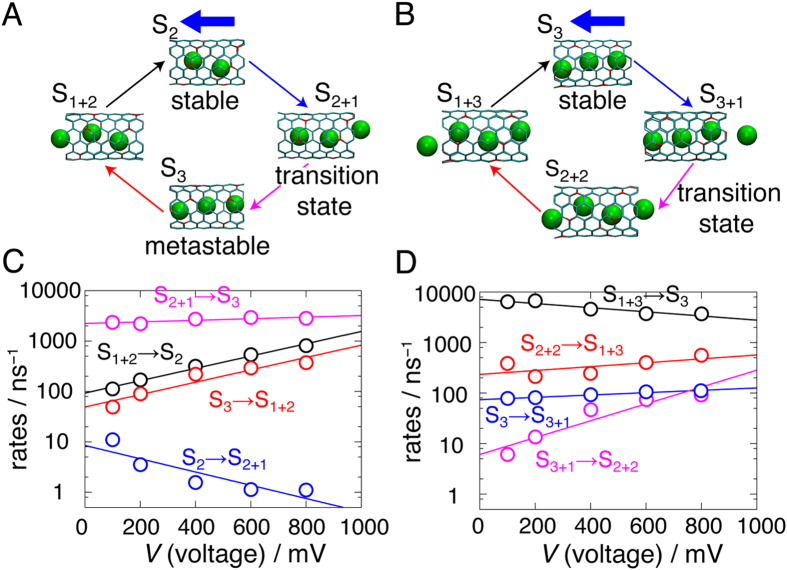
Ion permeation scheme and rate constants. (**A**) A sequence of states representing ion permeation through ANT^−5^. Only ANT and the permeating ions (green spheres) are depicted for the sake of clarity. A blue thick arrow indicates the direction of the flow of ions. Thin arrows show the transitions among states. Ion permeation occurs via the following sequence of states: S_2_ → S_2+1_ → S_3_ → S_1+2_ → S_2_. (**B**) A sequence of states representing ion permeation through ANT^−6^. Ion permeation through ANT^−6^ occurs via the following sequence of states: S_3_ → S_3+1_ → S_2+2_ → S_1+3_ → S_3_. (**C**) The voltage dependencies of rate constants among states in the case of ANT^−5^. The lines are the fitting curves by the exponential function, *k*_*ij*_(0)exp(*deV*/*k*_B_*T*). Fitted parameters for *k*_*ij*_(0) and *d* are indicated in Table 1. (D) The voltage dependencies of rate constants among states in the case of ANT^−6^. The lines are the fitting curves by the exponential function. Fitted parameters for *k*_*ij*_(0) and *d* are indicated in Table 1.

The permeation through the ANT can be described by a sequence of ion occupancy states (Fig. 2A,B). For ANT^−5^, an alternation of the stable (S_2_) and metastable (S_3_) states via intermediate states (S_1+2_ and S_2+1_) is equivalent to the transport of net one ion. The number of ions in ANT^−6^ is three, so the permeation sequence is S_3_ → S_3+1_ → S_2+2_ → S_1+3_ → S_3_.

The rate constant for the transition from the state *i* to the state *j* (*k*_*ij*_) was estimated by counting the number of the transition from the state *i* to the state *j* (*J*_*ij*_). The *k*_*ij*_ and *J*_*ij*_ have a following relation: *J*_*ij*_ = *k*_*ij*_*P*_*i*_, where *P*_*i*_ is the probability occupying the state *i*^19^. Evaluated voltage dependency of the rate constants is shown in Fig. 2C (ANT^−5^) and D (ANT^−6^). To a first approximation, the dependency of the rate constants on *V* was fitted by the exponential function; *k*_*ij*_(0)exp(*deV*/*k*_B_*T*), where *k*_B_ and *T* are the Boltzmann constant and the temperature, respectively^20^. *k*_*ij*_(0) and *d* are the rate constant at 0 mV and the so-called electrical distance, respectively^19^. Fitted values of *k*_*ij*_(0) and *d* are shown in Table 1.

It is found that *d* is sensitive to the energy difference (Δ*E*) for the process. For example, S_3_ → S_1+2_ and S_1+2_ → S_2_ through ANT^−5^ and S_3+1_ → S_2+2_ through ANT^−6^ are energy demanding processes, that is, Δ*E* is positive^18^. These processes are noticeably accelerated by adding voltage, thus the values of *d* in these processes are positive. On the other hand, others are the processes in which the energy decreases or is mostly conserved (Δ*E* is well below the thermal energy, *k*_B_*T*)^18^. *d* of those processes are nearly zero (*d* < 0.025) or negative.

In the case of ANT^−5^, the rate from S_2_ to S_2+1_ is the lowest, confirming that this process is indeed rate-limiting and S_2+1_ is the transition state^18^. This process is entropy bottlenecked and has negative Δ*E*^18^, so *d* in the process is negative. Thus, the rate of this process is not accelerated by *V*: especially, it is actually independent of *V*, when *V* is larger than 600 mV. This limits the current at high *V*, leading to the saturation of the current. This is an alternative interpretation of sublinear *i-V* curves, replacing the conventional explanation via the diffusion limitation.

By contrast, for ANT^−6^, the rate from S_3+1_ to S_2+2_ is the lowest; the ion proceeding into ANT against the ion-ion repulsion after the arrival at the entrance is rate-determining. The applied voltage decreases the barrier arisen from the repulsion and thus accelerates the rate of this process. This is the reason why the *i-V* curve through ANT^−6^ is superlinear. When *V* > 400 mV, the rate from S_3+1_ to S_2+2_ becomes close to that from S_3_ to S_3+1_. This confirms that the transition state is located in between S_3+1_ and S_2+2_ at 384 mV^18^.

The above two cases clarify the physical meaning of the shape of the *i-V* curves at two extreme limits: the *i-V* curve is sublinear or saturated when the free energy barrier for ion permeation mainly arises from entropic contribution, even though the permeation is not diffusion limited. On the other hand, the *i-V* curve is superlinear when the barrier primarily derives from energetic contribution.

### The meaning of the *i*-*V* curves through the K^+^ channels

It is interesting to see the *i-V* curves through the K^+^ channels: one example is that through the Kv1.2 channel. The experimentally measured curve was nearly linear below 90 mV^21^. However, the *i-V* curve through the Kv1.2 channel calculated by Jensen *et al*. is, as noted above, superlinear with zero current below the physiological voltage (<200 mV). The comparison of the *i-V* curves between the experiment and the MD simulation was given in Fig. 2 in the reference 15. Our result implies that the K^+^ fluxes in the simulation are limited by the barrier originated from the energy term; Δ*E* for the ion entering into the narrowest part of the channel is positive. The voltage of 200 mV, corresponding to the energy difference of 4.6 kcal/mol, was too low to drive ions to surmount the barrier, thus ions did not permeate. Therefore, the MD simulation performed by Jensen *et al*. has likely failed to capture the rate-limiting step, at least to reproduce the components of the free energy barrier.

**Figure 3:**
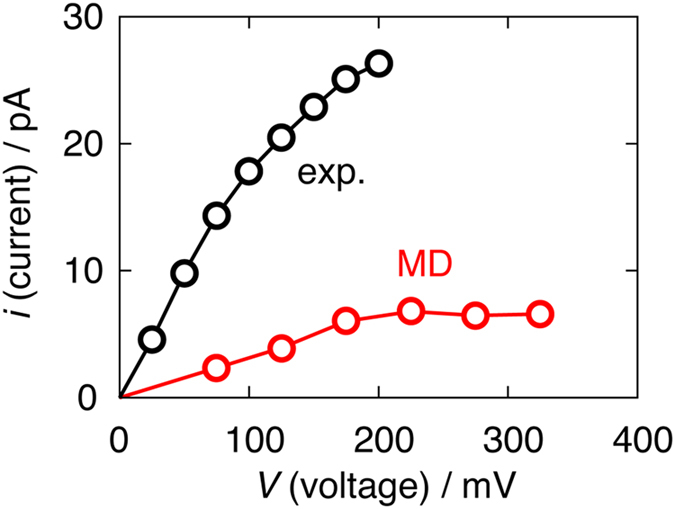
The current-voltage (*i-V*) curve through the KcsA channel at 400 mM evaluated by analyzing the permeation data obtained by the molecular dynamics simulation done by Köpfer *et al*. (Fig. 1D in their paper) (red line)^16^. The current and voltage are estimated from the time taking in the permeation of one ion and from the color bar showing the voltage at the relevant time, respectively. The average current and voltage obtained in this analysis are 5.8 pA and 227 mV, which are close to the reported values in the paper (5.3 pA and 210 mV). The experimentally measured *i-V* curve through the KcsA channel at 400 mM (shown in the reference 22) is also indicated in black.

The other example is the K^+^ flux through the KcsA channel, where the experimentally measured *i-V* curve was sublinear^22^. Köpfer *et al*. observed the ion permeation using the MD simulation under fluctuating voltage, allowing us to roughly estimate the *i-V* curve^16^. Köpfer *et al*. did not show the *i-V* curve in their paper, so it was evaluated here. Figure 3 shows the *i-V* curve through the KcsA channel at 400 mM obtained by analyzing the Köpfer’s trajectories (in red). The experimental result^22^ evaluated by LeMasurier *et al*. is also indicated (in black). The shape of the *i-V* curve obtained by the simulation is sublinear, reproducing the experimental behavior, but the current amplitudes are about one-fifth of the experimental ones.

Köpfer *et al*. discussed that the high throughput rates of the permeation can be explained by the direct knock-on mechanism, where the ions in the channel are pushed by an entering ion^16^. This process would have an energy barrier (a large Δ*E*) due to the ion-ion repulsion. If the transport is controlled by the direct knock-on, the *i-V* curve is expected to be superlinear based on this study. The sublinear *i-V* curve seen in Fig. 3 (and also in the experiment) thus indicates that the knock-on process does not control the overall permeation rate (or current). At the same time, they also suggested that the permeation through the channel is diffusion-limited. However, two issues should be pointed out: first, according to Table S1 in the paper, though the concentration (*C*) decreases to 200 mM, the current does not change. Second, five ion permeation events occurred in their Movie S1, while ~40 ions diffused to the entrance of the channel. These facts strongly indicate that the diffusion does not control the current. Therefore, the ion permeation simulated by Köpfer *et al*. is governed by the process which is irrelevant to *V* and *C*, at least under the condition of *V* > 200 mV and *C* > 200 mM.

Based on the present study, the sublinear curve implies that, even with an increase in *V*, there is a little impact in the acceleration of the permeation rate, therefore, it is determined by the process such as that of the exchange. Indeed, in their permeation scheme, K^+^ ions entered the narrowest part of the channel (called the selectivity filter; SF, in which ions line in a single-file), only after a water molecule already located in the SF left toward the space that ions come from^16^. In other words, the replacement of a water molecule in the SF with an incoming ion is necessary for ion permeation, like through ANT^−5^. If this exchange is rate-determining, the *i-V* curve would be sublinear. For ANT^−5^, the incoming ion coordinates to the water molecule coordinating to the ion in ANT^17^,^18^. As a result, water and ions permeate alternately. On the other hand, in the case of the simulation of Köpfer *et al*., the incoming ion exchanges position with the water molecule coordinating to the ion in the channel, causing no water permeation^16^. It must be mentioned that, however, this result is remarkably different from the experimental data, clarifying that approximately one water molecule is transported per each ion permeation across the SF^23^,^24^,^25^,^26^,^27^.

## Conclusion

We here demonstrated that the *i-V* curve reflects the physical origin of the free energy barrier determining the permeation rate or current through the nanopore; whether the rate-limiting process is accelerated by the voltage or not. ANT^−5^ having the entropy bottlenecked rate-limiting step showed the sublinear *i-V* curve, while ANT^−6^ possessing the energy bottlenecked one indicated the superlinear *i-V* curve. Note that the sublinearity of the curve does not necessarily mean that the permeation is diffusion-limited as opposed to the past permeation theory^7^,^10^. The configurational restraint on ions and water molecules when they line in a single-file at the narrowest parts of the pore would result in the sublinear *i-V* curves. On the other hand, when *i-V* curves are superlinear, a process surmounting the energetic barrier such as the knock-on mechanism^6^ could be critical for determining the current. Thus, the shape of the *i-V* curve is a good indicator to explore the microscopic mechanism of ion transport.

It is found that the fact that most biological channels show the sublinear *i-V* curves suggests that the currents through them are entropy bottlenecked. A few examples that indicates the superlinear *i-V* curve are the ion permeation through small peptide channels^28^,^29^,^30^ and the Rb^+^ permeation through the KcsA K^+^ channel^22^. Due to the small sample number, the origin of the superlinearity has been hardly clarified yet, but it has been considered that the *i-V* curve is superlinear when the rate-limiting step is the ion translocation across the channel^31^, or the channel is thought to transition from single to double ion occupancy when the *i-V* curve changes from sub- to superlinear^29^,^32^. Our result suggests an interpretation concerning the latter: the applied voltage will compensate the repulsion between the double ions likely participating in the knock-on process that does not exist when the channel is occupied by a single ion, thus the *i-V* curve becomes superlinear in the case of double occupancy.

Recently, current rectification through nanopores has been reported^33^,^34^,^35^. Some of them indicate even diode-like *i-V* curves^33^,^34^. The present study might suggest an alternative design for a diode: nanopores with asymmetric structures yielding an energy barrier at one end and an entropic barrier at the other end are the potential candidates for such diodes, since such nanopores would show superlinear *i-V* curve from one side and sublinear *i-V* curve from the other side.

One of the most sophisticated and modern techniques to observe the ion permeation through the biological channels is the MD simulation. Unfortunately, the MD simulation performed by Jensen *et al*. has failed to reproduce the shape of the *i-V* curve obtained by experiment^15^. Also, the direct knock-on mechanism suggested by Köpfer *et al*. was found not likely to give a rational explanation for the *i-V* curve that they simulated^16^. The difference in the *i-V* curve between ANT^−5^ and ANT^−6^ suggests why it is difficult to reproduce the *i-V* curve using the MD simulations: the charge difference of 1*e* completely altered the sublinear curve to the superlinear one. Additionally, in the previous papers^17^,^18^, it was found that the change of the charge of 0.4*e*, not as large as 1*e*, is enough to alter the permeation mechanism. Since the K^+^ channels are tetramer, a slight change of the parameter, corresponding to only 0.1*e* per monomer, will alter the *i-V* curve. In this work, the fixed-charge model was used, that is, the quantum effects such as the charge transfer^36^,^37^ or the polarization^38^ were not taken into account. Such effects could be as effective as the change of the charge of 0.1*e* per monomer. Also, these effects were shown to be critical to a subtle balance between the energetic and entropic contributions: the amount of energetic destabilization was found to be reduced when the quantum effects are included^39^. This reduction of the energy barrier would contribute to the sublinear *i-V* curve, which is observed through most channels. Therefore, in order to study the atomistic details of the ion permeation through the channels, tuning-up of the potential field to implicitly include the quantum effects or explicitly include them is inevitable to reproduce the experimentally measured *i-V* curve. Such improvements of the potential parameter will be also rewarding to promote protein science.

## Methods

The system consists of 24 K^+^ ions, 1106 water molecules, eleven CNTs, and one ANT, corresponding to the bulk concentration of ~1 M. The CNTs correspond to membrane in the present simulation, since K^+^ ions never get into them and only permeate through the ANT. The negative charges are helically arranged on ANT (the detailed configuration of negative charges is given in the reference 17). The TIP3P model^40^ and AMBER default parameter^41^ were used for water molecules and K^+^ ions, respectively. AMBER94 *sp*^2^ carbon^41^ was used for carbon atoms in CNTs and ANT. The equilibrium bond length between carbon atoms was shortened from 1.4 to 1.2 Å to mimic the length of the selectivity filter of the K^+^ channels. Two kinds of negative charge on ANT were examined; total amounts of charge are −5.0*e* or −6.0*e*, where *e* is the elementary charge.

MD simulations were performed at constant volume and temperature of 300 K using Berendsen’s thermostat^42^. The periodic boundary condition with the size of 31.306 × 36.098 × 43.010 Å^3^ was imposed. Long range interactions were calculated by the particle mesh Ewald method with an 11 Å real space cutoff^43^. The intramolecular coordinates in water molecule were constrained with the SHAKE algorithm^44^, enabling a time step of 2 fs.

To mimic the voltage-clamp measurement^45^, the passive transport of ions was realized by applying an electric field along the channel axis in the region of the ANT and CNTs^17^. The sander module of the AMBER package was modified to impose the electric field. The translation of ANT caused by the electric field was constrained by the harmonic potential with the force constant of 0.1 kcal/mol/Å^2^. Five voltages were applied: 100, 200, 400, 600, and 800 mV. At a certain condition, 100 different initial configurations were generated and were time-evolved for 10 ns, resulting in the simulation time of 1.0 μs. The ion trajectories were saved at every 10 fs. To calculate currents at each voltage, the following equation was used: *eN*_ion_/*T*_sim_, where *N*_ion_ and *T*_sim_ are the number of ions that entered the ANT at a certain condition and the simulation time at the condition, respectively. Since an ion entering from one side and other ion’s exiting to the other side is tightly coupled^17^,^18^, *N*_ion_ is almost the same as the number of ions exiting from the ANT.

## Additional Information

**How to cite this article**: Sumikama, T. Origin of the Shape of Current-Voltage Curve through Nanopores: A Molecular Dynamics Study. *Sci. Rep.* **6**, 25750; doi: 10.1038/srep25750 (2016).

## Figures and Tables

**Table 1:** Rate constants at 0 mV (*k*_*ij*_(0)) and electrical distances (*d*).

*i* → *j*	ANT^−5^				ANT^−6^			
	S_2_ → S_2+1_	S_2+1_ → S_3_	S_3_ → S_1+2_	S_1+2_ → S_2_	S_3_ → S_3+1_	S_3+1_ → S_2+2_	S_2+2_ → S_1+3_	S_1+3_ → S_3_
*k*_*ij*_(0)	8.5	2230.6	49.1	93.0	73.9	6.1	234.3	7151.1
*d*	−0.079	0.009	0.074	0.073	0.014	0.100	0.023	−0.025

The unit of *k*_*ij*_(0) is ns^−1^.
